# Supporting Trans and Non-Binary People – Best Practice Guidance for Health & Wellbeing Practitioners – a Bristol-Based Project Co-Produced by Mental Health Specialists, a Charitable Organisation Stand Against Racism & Inequality (SARI), Local LGBTQ+ Champions and the Local Trans and Non-Binary Community

**DOI:** 10.1192/bjo.2025.10280

**Published:** 2025-06-20

**Authors:** Daniel Hodgson, Alex Raikes

**Affiliations:** 1 AWP, Bristol, United Kingdom; 2 SARI, Bristol, United Kingdom

## Abstract

**Aims:** Mental health support for trans and non-binary people has been under scrutiny for many years. Trans and non-binary people often present to mental health services with co-morbid mental health difficulties. Medical practitioners often report a lack of training and understanding. GPs, psychiatrists and allied health professional frequently request more information to help educate and inform their practice and to support them in offering the best care for their patients and their families and carers.

**Methods:** A small working group was set up by AWP (Avon and Wiltshire Partnership NHS Trust) and SARI (a Bristol-based charitable organisation Stand Against Racism & Equality) with members of the trans and non-binary community in the South West of England. The group’s objectives were to develop novel training for staff and to produce some written guidance that reflected all the evidence-base. This guidance was identified as needing to be for education and guidance only rather than anything prescriptive. It also needed to be easy to read, accessible and available in different formats to reach a wide target audience.

**Results:** The working group was led by the trans and non-binary community and, in collaboration with the AWP, SARI and other health professionals, staff training and a guidance booklet was produced.

The training was aimed at providing health professionals with the knowledge and understanding to support gender diverse people that they may meet as patients, colleagues or friends. The training was unique in that it was the first training of its type to be written and delivered by trans and non-binary people. It has been a huge success and the feedback has been consistently outstanding.

The written guidance includes a booklet as an education tool for all health professionals. It is for guidance and understanding only rather than anything prescriptive. This was co-written by health professionals and the trans/non-binary community. The guidance includes important information about how to support a trans or non-binary person, common questions and misconceptions, the latest information about trans health care, and the interface with mental health services. This is particularly important in the current political climate where trans and non-binary people are feeling that their health care needs are threatened and that they are feeling increasingly vulnerable. It is important for psychiatrists, GPs and other health professionals to feel confident in supporting gender diverse people who may present to them experiencing significant mental health difficulties. This guidance addresses all the aspects of mental health co-morbidity and how to equip clinicians with the knowledge and skills to provide genuine care.

**Conclusion:** The training has been an enormous success and has attracted attention and positive feedback. Staff report that they now feel equipped with skills to support trans and non-binary people as patients, colleagues or in their personal lives. The fact that the training was delivered by trans and non-binary people has been described as unique and trail-blazing. Members of the trans community are also delighted to feel included and listened to.

The guidance booklet: Supporting Trans and Non-Binary People – Best Practice Guidance for Health & Wellbeing Practitioners was launched at various Pride events in the South West and shared with health professionals working in GP surgeries, mental health services and charitable organisations. The guidance itself has been praised widely reflected by a number of awards including AWP staff awards (winner) and the National Diversity Awards (finalist). The guidance has been cited as an essential education and guidance tool. It is updated regularly to reflect service changes and continues to be in high demand. This is something to be celebrated in this current climate where equality, diversity and inclusion remain central in how we support and look after our patients and each other.

(Copies of the booklet will be available to take away or can be accessed via the QR code on the day).

